# Decreased influenza vaccination coverage among Chinese healthcare workers during the COVID-19 pandemic

**DOI:** 10.1186/s40249-022-01029-0

**Published:** 2022-10-08

**Authors:** Libing Ma, Xuan Han, Yuan Ma, Yuan Yang, Yunshao Xu, Di Liu, Weizhong Yang, Luzhao Feng

**Affiliations:** 1 School of Population Medicine and Public Health, Chinese Academy of Medical Sciences & Peking Union Medical College, Beijing, 100730 China; 2 Department of Respiratory and Critical Care Medicine, The Affiliated Hospital of Guilin Medical University, Guilin, Guangxi 541001 China; 3 “Breath Circles” Network Platform, Beijing, China

**Keywords:** Influenza, Healthcare worker, Vaccination, Coverage, Internet-based survey, China

## Abstract

**Background:**

Healthcare workers (HCWs) were the priority group for influenza vaccination, in China during the 2020/2021 and 2021/2022 influenza seasons. However, vaccination rates in HCWs have always been low. This study investigated influenza vaccination status among Chinese HCWs and analyzed the factors driving vaccination.

**Methods:**

We provided electronic questionnaires to HCWs from January 27, 2022 to February 21, 2022, using the WeChat platform "Breath Circles". HCWs who received the link could also forward it to their colleagues. Binary logistic regression models were used to analyze vaccination-associated factors among HCWs.

**Results:**

Among the 1697 HCWs surveyed, vaccination coverage was 43.7% (741/1697) during the 2020/2021 influenza season, and 35.4% (600/1697) during the 2021/2022 influenza season, as of February 21, 2022. Additionally, 22.7% (385/1697) and 22.1% (358/1697) of HCWs reported that their workplaces implemented a free vaccination policy for all employees during the 2020/2021 and 2021/2022 influenza seasons. HCWs who were required to be vaccinated according to hospital regulations, and whose hospitals implemented the free influenza vaccine policy were more likely to be vaccinated (2020/2021 and 2021/2022; *P* < 0.05). In addition, the economic level of the HCWs' province (2021/2022, *P* < 0.05) and the HCWs’ knowledge about vaccination and willingness to get vaccinated, such as active learning about vaccines (2020/2021, *P* < 0.05), supportive attitude toward vaccination for all HCWs (2020/2021 and 2021/2022; *P* < 0.05), also had an impact on vaccine coverage.

**Conclusions:**

A free influenza vaccination policy and workplace required vaccination are effective in improving influenza vaccination coverage among HCWs. Influenza vaccination coverage of Chinese HCWs remained low and showed a downward trend after the COVID-19 outbreak. Further effective measures, such as advocacy campaigns, free vaccine policies, and on-site vaccination could be implemented to improve influenza vaccination coverage.

**Graphical abstract:**

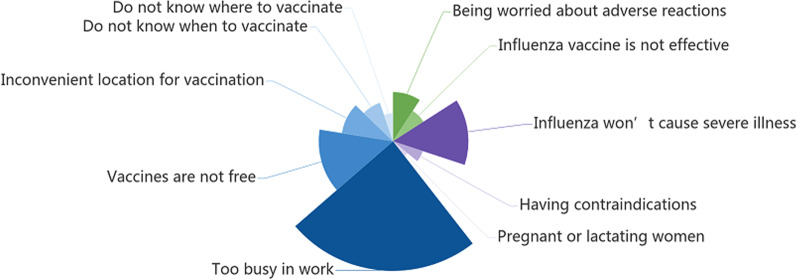

**Supplementary Information:**

The online version contains supplementary material available at 10.1186/s40249-022-01029-0.

## Background

Seasonal influenza is a serious respiratory infectious disease caused by influenza virus. Influenza contributes to 291,243–645,832 deaths from respiratory-related diseases worldwide annually, as estimated in 2018 [1]. Nonetheless, existing studies have focused on influenza-related respiratory mortality, which may underestimate the true burden of death caused by influenza [1].

Vaccination remains the most effective way to reduce the incidence and severity of influenza [2]. It reduces the risk of contracting influenza and alleviates influenza symptoms, thereby significantly reducing the burden of disease and mortality. However, strains mutate rapidly and the protective effect after vaccination lasts approximately 6 months; therefore, annual influenza vaccination alone guarantees a preventive effect [3].

Due to their occupation, HCWs are at a higher risk of exposure to respiratory pathogens than the general population [4]. Infected HCWs may cause epidemics in hospitals by spreading the disease to patients and their families, as well as to their family members. Unvaccinated HCWs have higher absenteeism than vaccinated HCWs, which leads to direct and indirect costs [5]. The willingness and behavior of HCWs toward influenza vaccination are critical and need to be evaluated. This study aimed to determine the coverage of influenza vaccination, identify the factors driving vaccination among Chinese HCWs during the 2020/2021 and 2021/2022 influenza seasons, understand the impact of the COVID-19 pandemic on vaccination coverage, and suggest effective measures to increase influenza vaccination coverage among HCWs.

## Methods

### Study design

From January 27, 2022 to February 21, 2022, an internet-based cross-sectional study was conducted on the WeChat platform "Breath Circles" (https://mp.weixin.qq.com/s/xJlQ3ifK2tw_D30Dt0S91A). WeChat is a free application that provides instant messaging services for smart terminals, reaching more than 94% of smartphones in China, which can be linked to many "third-party applets". Our respondents were users of the "Breath Circle" applet, a platform integrates and publishes authoritative information on respiratory medicine. The user group consists mainly of respiratory medicine professionals. As of April 19, 2022, the total number of users reached 235,000, covering 29 provincial-level administrative divisions (PLADs) in China. The eligibility criteria required the users to specify their occupation and confirm that they were working in a hospital. Based on our previous studies, a questionnaire (Additional file 1) consisting of three main aspects was designed: (1) Basic details of the HCWs, including age, gender, and occupation. (2) Workplace policies regarding influenza vaccination. (3) HCWs’ knowledge about vaccination and willingness to get vaccinated, reasons for hesitation, and suggestions for expanding vaccination coverage. The questionnaire was distributed to "Breath Circles" users in the form of questionnaire star(wjx.cn), a questionnaire design program.

### Data collection

We posted a link to the questionnaire on the “Breath Circles” forum so that HCWs who received the link could forward it to their colleagues; each participant could only response once. For some questions, the respondents could select multiple options. According to the National Influenza Prevention and Control Plan of the Chinese government, we divided the departments into two groups: "high-risk departments" included respiratory, infectious diseases, emergency, pediatrics, intensive care unit/intensive medicine, fever clinic, geriatrics, and obstetrics and gynecology departments; and "other departments" included the remaining departments. We obtained demographic data from the National Health Commission of China to compare our study population with the general population of Chinese HCWs in 2021 [6], (Additional file 2: Table S1). The gross domestic product (GDP) per capita of the PLADs was obtained from the National Bureau of Statistics [7].

Our definitions of vaccination policies and workplace regulations were as follows:

1. Vaccination policies were divided into four types: (i) the hospital offered free influenza vaccination to all hospital employees, (ii) the hospital offered free vaccination to personnel in high-risk department, (iii) the hospital offered no free influenza vaccine to employees, and (iv) the respondent were unsure of the hospital’s policy. The free vaccination policy meant that the cost of vaccines and vaccination services was covered by the workplace, including direct payment or reimbursement after vaccination.
2. There were four types of vaccination regulations in the workplace: required, encouraged, no intervention, and unknown. Required vaccinations: hospitals issue official documents or regulations requiring employees to be vaccinated; encouraged vaccinations: hospitals incentivize employees to receive the flu vaccine through health education or dissemination of knowledge; no intervention: hospitals neither required nor encouraged employees to be vaccinated; unknown: respondents were unaware of any workplace regulations regarding influenza vaccines.

### Quality control

Based on expert consultation, a unified survey plan and questionnaire were developed and improved. Before the survey, precautions were communicated in depth with the person in charge of the “Breath Circle” platform. The questionnaire was distributed through the online link, and the date of completion date was restricted. After collecting questionnaires, those with missing important and obvious logical errors were excluded. Valid survey respondents with complete basic information and influenza vaccination status in the 2020/2021 and 2021/2022 influenza seasons and those working in hospitals were included in the analysis.

### Statistical analysis

Survey results from the questionnaire star were imported into Microsoft Excel version 2016 (Microsoft Corporation, Redmond, USA) for data collation and cleaning, and analyzed with the SPSS Statistics software version 24 (SPSS Inc., Chicago, USA). All categorical variables were compared using the chi-square test (α = 0.05). Binary logistics regression models were used to analyze the factors associated with vaccination among HCWs. The dependent variable was whether the HCW was vaccinated during the 2020/2021 and 2021/2022 influenza seasons. Demographics, workplace vaccination policies, and vaccination cognition, and willingness were included in the regression models as independent variables.

## Results

### Demographics of the study population

This survey collected 1697 valid questionnaires. Of the 1697 respondents, 187 (11.0%) worked in primary hospitals, 392 (23.1%) in secondary hospitals, and 1118 (65.9%) in tertiary hospitals; with 1095 (64.5%) in high-risk departments and 602 (35.5%) in other departments. There were 1023 clinicians (60.3%), 438 nurses (25.8%), 21 vaccinators (1.2%), 104 medical technicians (6.1%), and 111 respondents in other categories (6.5%) (Table 1). The median age of the surveyed HCWs was 37 years (range 18–65 years) and the median experience duration was 12 years (range < 1 to 45 years).

**Table 1:** Respondent characteristics and influenza vaccination status, *n* (%)

Characteristics	Total	Influenza vaccination during the 2020/2021 influenza season		Influenza vaccination during the 2021/2022 influenza season	
		Yes	No	Yes	No
Total	1697	741(43.7)	956(56.3)	600(35.4)	1097(64.6)
Demographics of HCWs					
Age, years		*P* = 0.005		*P* = 0.04	
< 25	105	45 (42.9)	60 (57.1)	42 (40.0)	63 (60.0)
25–34	553	206 (37.3)	347 (62.7)	167 (30.2)	386 (69.8)
35–44	683	311 (45.5)	372 (54.5)	248 (36.3)	435 (63.7)
45–54	287	145 (50.5)	142 (49.5)	117 (40.8)	170 (59.2)
55–59	52	26 (50.0)	26 (50.0)	20 (38.5)	32 (61.5)
≥ 60	17	8 (47.1)	9 (52.9)	6 (35.3)	11 (64.7)
Degree		*P* = 0.69		*P* = 0.64	
≤ Technical secondary school	30	15 (50.0)	15 (50.0)	13 (43.3)	17 (56.7)
Bachelor & Junior college student	1267	556 (43.9)	711 (56.1)	448 (35.4)	819 (64.6)
Postgraduate	400	170 (42.5)	230 (57.5)	139 (34.8)	261 (65.3)
PLAD by GDP per capita^a^		*P* < 0.0001		*P* < 0.0001	
Low GDP	1146	468 (40.8)	678 (59.2)	369 (32.2)	777 (67.8)
Middle GDP	318	140 (44.0)	178 (56.0)	111 (34.9)	207 (65.1)
High GDP	233	133 (57.1)	100 (42.9)	120 (51.5)	113 (48.5)
Occupation		*P* < 0.0001		*P* < 0.0001	
Clinician	1023	428 (41.8)	595 (58.2)	335 (32.7)	688 (67.3)
Nurse	438	202 (46.1)	236 (53.9)	175 (40.0)	263 (60.0)
Medical technician^b^	104	56 (53.8)	48 (46.2)	48 (46.2)	56 (53.8)
Vaccination staff	21	17 (81.0)	4 (19.0)	13 (61.9)	8 (38.1)
Others	111	38 (34.2)	73 (65.8)	29 (26.1)	82 (73.9)
Years of working		*P* = 0.01		*P* = 0.09	
≤ 5	300	115 (38.3)	185 (61.7)	101 (33.7)	199 (66.3)
5–9	325	126 (38.8)	199 (61.2)	99 (30.5)	226 (69.5)
10–19	618	277 (44.8)	341 (55.2)	219 (35.4)	399 (64.6)
20–29	319	155 (48.6)	164 (51.4)	125 (39.2)	194 (60.8)
≥ 30	135	68 (50.4)	67 (49.6)	56 (41.5)	79 (58.5)
Hospital category^c^		*P* = 0.054		*P* = 0.26	
Primary hospitals	187	79 (42.2)	108 (57.8)	57 (30.5)	130 (69.5)
Secondary hospitals	392	192 (49.0)	200 (51.0)	147 (37.5)	245 (62.5)
Tertiary hospitals	1118	470 (42.0)	648 (58.0)	396 (35.4)	722 (64.6)
Department		*P* = 0.93		*P* = 0.99	
High risk departments	1095	479 (43.7)	616 (56.3)	387 (35.3)	708 (64.7)
Other departments	602	262 (43.5)	340 (56.5)	213 (35.4)	389 (64.6)
Professional title		*P* = 0.03		*P* = 0.18	
Above intermediate	450	186 (41.3)	264 (58.7)	155 (34.4)	295 (65.6)
Intermediate	612	270 (44.1)	342 (55.9)	218 (35.6)	394 (64.4)
Below intermediate	536	253 (47.2)	283 (52.8)	201 (37.5)	335 (62.5)
Unclassified/unknown	99	32 (32.3)	67 (67.7)	26 (26.3)	73 (73.7)
Whether the Hospital has set up a routine vaccination clinic		*P* < 0.0001		*P* < 0.0001	
Yes	1279	611 (47.8)	668 (52.2)	496 (38.8)	783 (61.2)
No	418	130 (31.1)	288 (68.9)	104 (24.9)	314 (75.1)
Whether daily work involve in vaccination work		*P* < 0.0001		*P* < 0.0001	
Yes	637	331 (52.0)	306 (48.0)	285 (44.7)	352 (55.3)
No	1060	410 (38.7)	650 (61.3)	315 (29.7)	745 (70.3)
Workplace vaccination policies					
Workplace’s policy		*P* < 0.0001		*P* < 0.0001	
Requirement^d^	188	139 (73.9)	49 (26.1)	124 (66.0)	64 (34.0)
Promotion	955	515 (53.9)	440 (46.1)	433 (45.3)	522 (54.7)
None	428	80 (18.7)	348 (81.3)	42 (9.8)	386 (90.2)
Not clear	126	7 (5.6)	119 (94.4)	1 (0.8)	125 (99.2)
Free vaccination		*P* < 0.0001		*P* < 0.0001	
For all staff	518	385 (74.3)	133 (25.7)	358 (69.1)	160 (30.9)
For high-risk department	258	158 (61.2)	100 (38.8)	124 (48.1)	134 (51.9)
Have not free vaccination policy	740	161 (21.8)	579 (78.2)	100 (13.5)	640 (86.5)
Not clear	181	37 (20.4)	144 (79.6)	18 (9.9)	163 (90.1)
HCWs’ knowledge about vaccination and willingness to get vaccinated					
Whether taken the initiative to learn about vaccines and health related knowledge		*P* < 0.0001		*P* < 0.0001	
Yes	1421	658 (46.3)	763 (53.7)	541 (38.1)	880 (61.9)
No	276	83 (30.1)	193 (69.9)	59 (21.4)	217 (78.6)
Frequency of learning vaccines and health-related knowledge		*P* < 0.0001		*P* < 0.0001	
Once a day	84	65 (77.4)	19 (22.6)	54 (64.3)	30 (35.7)
Once a week	398	207 (52.0)	191 (48.0)	177 (44.5)	221 (55.5)
Once a month	526	226 (43.0)	300 (57.0)	184 (35.0)	342 (65.0)
Once half of a year	285	114 (40.0)	171 (60.0)	91 (31.9)	194 (68.1)
Once a year	128	46 (35.9)	82 (64.1)	35 (27.3)	93 (72.7)
None	276	83 (30.1)	193 (69.9)	59 (21.4)	217 (78.6)
Frequency of recommending respiratory infectious diseases’ vaccine to suitable populations		*P* < 0.0001		*P* < 0.0001	
Frequently	899	491 (54.6)	408 (45.4)	402 (44.7)	497 (55.3)
Occasionally	685	234 (34.2)	451 (65.8)	188 (27.4)	497 (72.6)
Never	113	16 (14.2)	97 (85.8)	10 (8.8)	103 (91.2)
Whether support all HCWs to uptake influenza vaccine		*P* < 0.0001		*P* < 0.0001	
Yes	1510	719 (47.6)	791 (52.4)	585 (38.7)	925 (61.3)
No	187	22 (11.8)	165 (88.2)	15 (8.0)	172 (92.0)
Whether uptake influenza vaccine if the vaccination is free		*P* < 0.0001		*P* < 0.0001	
Yes	1479	712 (48.1)	767 (51.9)	585 (39.6)	894 (60.4)
No	77	8 (10.4)	69 (89.6)	7 (9.1)	70 (90.9)
Not clear	141	21 (14.9)	120 (85.1)	8 (5.7)	133 (94.3)

^a^In terms of GDP per capita, PLADs are divided into three levels: low, middle and high. Low for Anhui, Qinghai, Jiangxi, Shanxi, Tibet, Heilongjiang, Guangxi, Guizhou, Yunnan, Gansu; Middle for Chongqing, Shaanxi, Liaoning, Jilin, Ningxia, Hunan, Hainan, Henan, Xinjiang, Sichuan, Hebei; High for Beijing, Shanghai, Tianjin, Jiangsu, Zhejiang, Fujian, Guangdong, Shandong, Inner Mongolia, Hubei

^b^Medical technicians include inspection, imaging, ultrasound, electrocardiogram, pharmacy, etc. Others include administration, logistics personnel, medical school staff, scientific research institute staff, medical students, etc.

^c^Primary hospitals: mainly refer to rural township hospitals and community health service centers that provide prevention, treatment, healthcare, and rehabilitation services directly to communities of a certain population in China. Secondary hospitals: mainly refer to county-level hospitals that provide comprehensive medical and health-care services to multiple communities and undertake certain teaching and scientific research tasks. Tertiary hospitals: hospitals above the regional level that provides high-level specialized medical and health-care services and carries out higher education and scientific research tasks to multiple regions

^d^Requirement means hospitals issued official document or regulation to ask employees to get compulsory vaccination, but HCWs who have not received influenza vaccination will not be punished

*GDP* Gross domestic product; *HCWs* healthcare workers; *PLADs* Provincial-level administrative divisions

*P* value from Chi-square test

### Implementation of free vaccination policies

Among the 1697 respondents, vaccination coverage was 43.7% (741/1697) and 35.4% (600/1697) in the 2020/2021 and 2021/2022 influenza seasons, respectively. Of the vaccinated HCWs in the 2021/2022 influenza season, 48.3% (290/600) received instant free vaccination, 14.8% (89/600) were reimbursed by hospitals after vaccination, 30.2% (181/600) were vaccinated at their own expense, 6.0% (36/600) received medicare reimbursement, and 0.7% (4/600) were paid by other means (Table 1).

### Influenza vaccination coverage among HCWs and associated factors

#### Factors associated with influenza vaccination

A summary of the factors influencing influenza vaccination coverage of HCWs during the 2020/2021 and 2021/2022 influenza seasons is shown in Table 2. The vaccination coverage among HCWs living in PLADs with medium per capita GDP was higher than that among HCWs living in PLADs with lower GDP per capita (2020/2021, *P* < 0.05). Additionally, the vaccination coverage among HCWs living in PLADs with medium and high per capita GDP was higher than that among HCWs living in PLADs with low GDP per capita (2021/2022, *P* < 0.05). The vaccination coverage among HCWs who were engaged in vaccination drives was higher than that among HCWs who were not engaged in vaccination drives (2020/2021 and 2021/2022; *P* < 0.05). Hospitals that set up routine vaccination clinics had higher vaccination coverage than hospitals that did not (2020/2021, *P* < 0.05) (Table 2).

**Table 2:** Factors associated with influenza vaccination

Variables (reference)	Flu vaccination during 2020/2021 influenza season (yes vs no)	Flu vaccination during 2021/2022 influenza season (yes vs no)
Demographics of HCWs		
Age (< 25 years)		
25–34	1.14 (0.65, 1.98)	
35–44	1.82 (1.03, 3.22)	
45–54	2.22 (1.20, 4.08)*	
55–59	1.88 (0.77, 4.57)	
≥ 60	2.43 (0.66, 8.93)	
PLAD by GDP per capita ^a^ (Low GDP)		
Middle GDP	1.55 (1.11, 2.17)*	1.63 (1.15, 2.29)*
High GDP	1.40 (0.94, 2.10)	1.65 (1.11, 2.45)*
Occupation (Clinician)		
Nurse	1.06 (0.78, 1.44)	1.16 (0.85, 1.58)
Medical technician^b^	1.45 (0.85, 2.47)	1.43 (0.83, 2.49)
Vaccination staff	6.55 (1.96, 21.85)*	4.53 (1.54, 13.33)*
Others	0.72 (0.41, 1.26)	0.70 (0.39, 1.25)
Whether the Hospital has set up a routine vaccination clinic (Yes)		
No	0.65 (0.48, 0.89)*	/
Whether daily work involve in vaccination work (Yes)		
No	/	0.71 (0.55, 0.93)*
Workplace vaccination policies		
Workplace’s policy (Requirement)^d^		
Promotion	0.63 (0.41, 0.95)*	0.72 (0.48, 1.06)
None	0.32 (0.19, 0.54)**	0.28 (0.17, 0.49)**
Not clear	0.09 (0.04, 0.23)**	0.03 (0.00, 0.19)**
Free vaccination (For all staff)		
For high-risk department	0.56 (0.38, 0.80)*	0.43 (0.31, 0.62)**
Have not free vaccination policy	0.13 (0.09, 0.19)**	0.11 (0.08, 0.16)**
Not clear	0.22 (0.13, 0.35)**	0.12 (0.07, 0.21)**
HCWs’ knowledge about vaccination and willingness to get vaccinated		
Whether taken the initiative to learn about vaccines and health related knowledge (Yes)		
No	0.42 (0.21, 0.84)*	
Frequency of learning vaccines and health-related knowledge (Once a day)		
Once a week	0.37 (0.2, 0.70)*	
Once a month	0.32 (0.17, 0.60)**	
Once half of a year	0.38 (0.19, 0.73)*	
Once a year	0.42 (0.19, 0.89)*	
None		
Frequency of recommending respiratory infectious diseases’ vaccine to suitable populations (Frequently)		
Occasionally	0.51 (0.39, 0.67)**	0.54 (0.41, 0.71)**
Never	0.24 (0.12, 0.48)**	0.20 (0.09, 0.43)**
Whether support all HCWs to uptake influenza vaccine (Yes)		
No	0.27 (0.16, 0.46)**	0.31 (0.16, 0.57)**
Whether uptake influenza vaccine if the vaccination is free (Yes)		
No	0.28 (0.12, 0.64)*	0.37 (0.15, 0.91)*
Not clear	0.42 (0.24, 0.75)*	0.18 (0.08, 0.40)**

Odds ratio and 95% confidence intervals were presented

*GDP* Gross domestic product; *HCWs* healthcare workers; *PLADs* Provincial-level administrative divisions

Significance level: **P < .01, *P < .05

#### Vaccination policies

HCWs who were required to be vaccinated according to hospital regulations were more likely to be vaccinated (2020/2021 and 2021/2022; *P* < 0.05), and HCWs whose hospital implemented the free influenza vaccine policy for all staff were also more likely to be vaccinated (2020/2021 and 2021/2022; *P* < 0.05).

#### Knowledge and willingness towards influenza vaccination

HCWs who actively learned about vaccines and were knowledgeable were more likely to be vaccinated than those who were not (2020/2021, *P* < 0.05). HCWs who supported vaccination for all HCWs were more likely to be vaccinated (2020/2021 and 2021/2022; *P* < 0.05). Moreover, if vaccination was provided for free, HCWs were more likely to be vaccinated (2020/2021 and 2021/2022; *P* < 0.05).

### Driving factors for influenza vaccination in the influenza season 2021/2022

Of the 600 HCWs who were vaccinated during the 2021/2022 influenza season, 69.5% of them got vaccinated out of concern for infecting others, 66.5% were worried about contracting the flu themselves, and 41.3% were concerned about the impact of influenza on their work (Table 3).

**Table 3:** Drivers of influenza vaccination among vaccinated healthcare workers (*n* = 600) in China, influenza season 2021/2022

Reasons for vaccination ^a^	*n*	Proportion (%)
Worry about spreading influenza to others	417	69.5
Worry about contacting influenza	399	66.5
Preventing/reducing absenteeism from work	248	41.3
Required by the workplace	217	36.2
Easy access to vaccination from the workplace	198	33.0
Recommendations from the national policy-making body (e. g. technical guidelines)	142	23.7
Free vaccination	87	14.5
Previous experience with vaccination	16	2.7

^a^These reasons are not mutually exclusive

### Barriers to influenza vaccination among HCWs in 2021/2022 influenza season

**Fig. 1:**
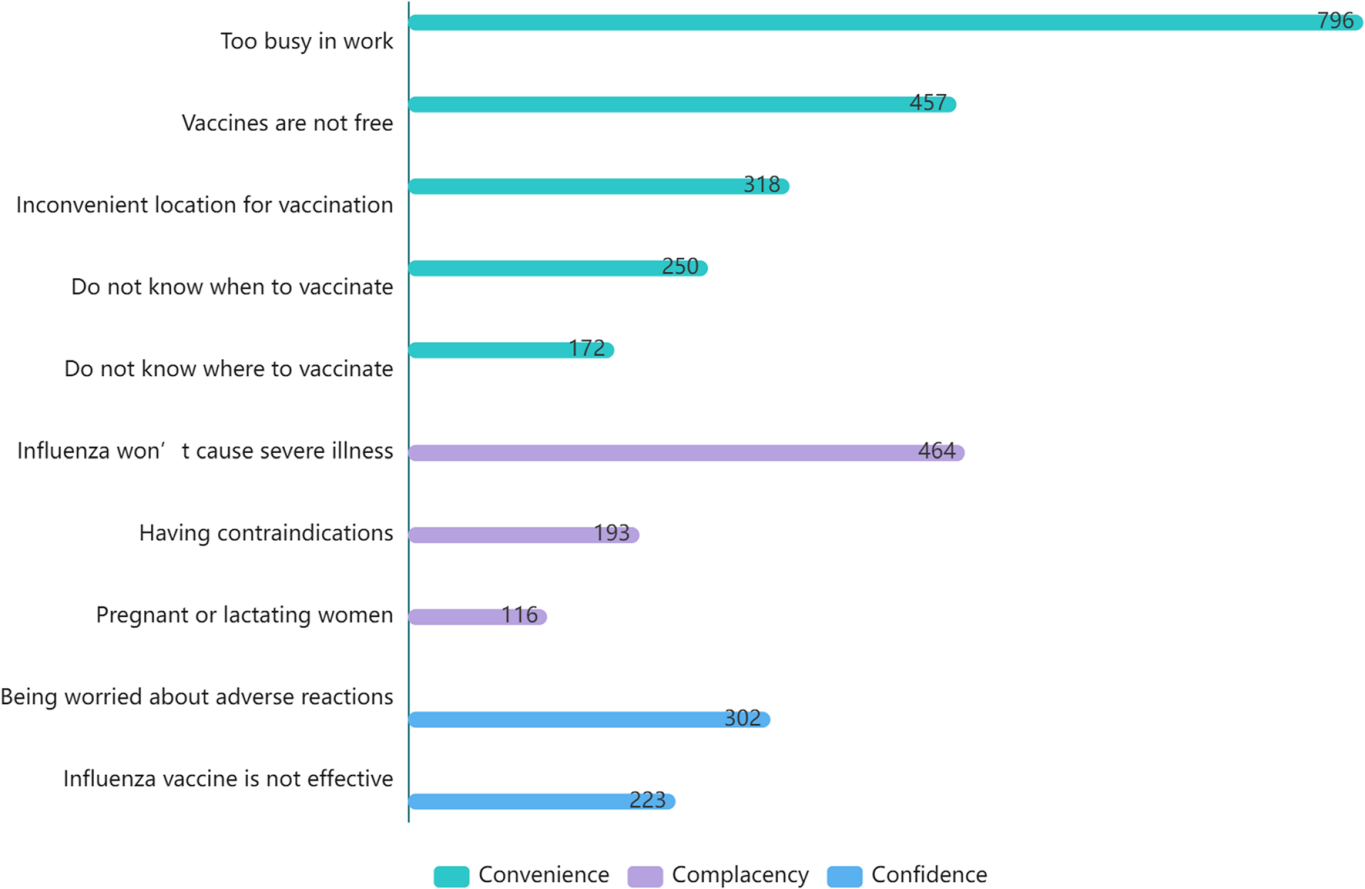
Barriers to influenza vaccination by healthcare workers in the 2021/2022 influenza season^ab^. ^a^The WHO uses the “3 Cs” model to classify vaccine hesitancy: confidence, complacency and convenience [8]. ^b^The x-axis represents the number of people who selected that option

Of the 1097 HCWs who were not vaccinated during the 2021/2022 influenza season, HCWs reported that the main reason for not getting vaccinated was being too busy at work (72.6%). Other reasons included the consideration that influenza infection was not serious (42.3%), reluctance to pay for vaccination (41.7%), inconvenient location for vaccination (29.0%), and fear of adverse reactions (27.5%) (Fig. 1).

### Factors supporting influenza vaccination among HCWs and the public

HCWs reported that the factor most likely to encourage influenza vaccination was the provision of free vaccination (82.1%). Other factors included setting up vaccination clinics or temporary vaccination points in the hospital (75.7%), encouragement by the hospital (61.6%), vaccination campaigns (58.5%), and requiring vaccination by the hospital (47.3%). The proportion of HCWs who believed that none of the above measures would prompt vaccination was only 4.8%.

**Fig. 2:**
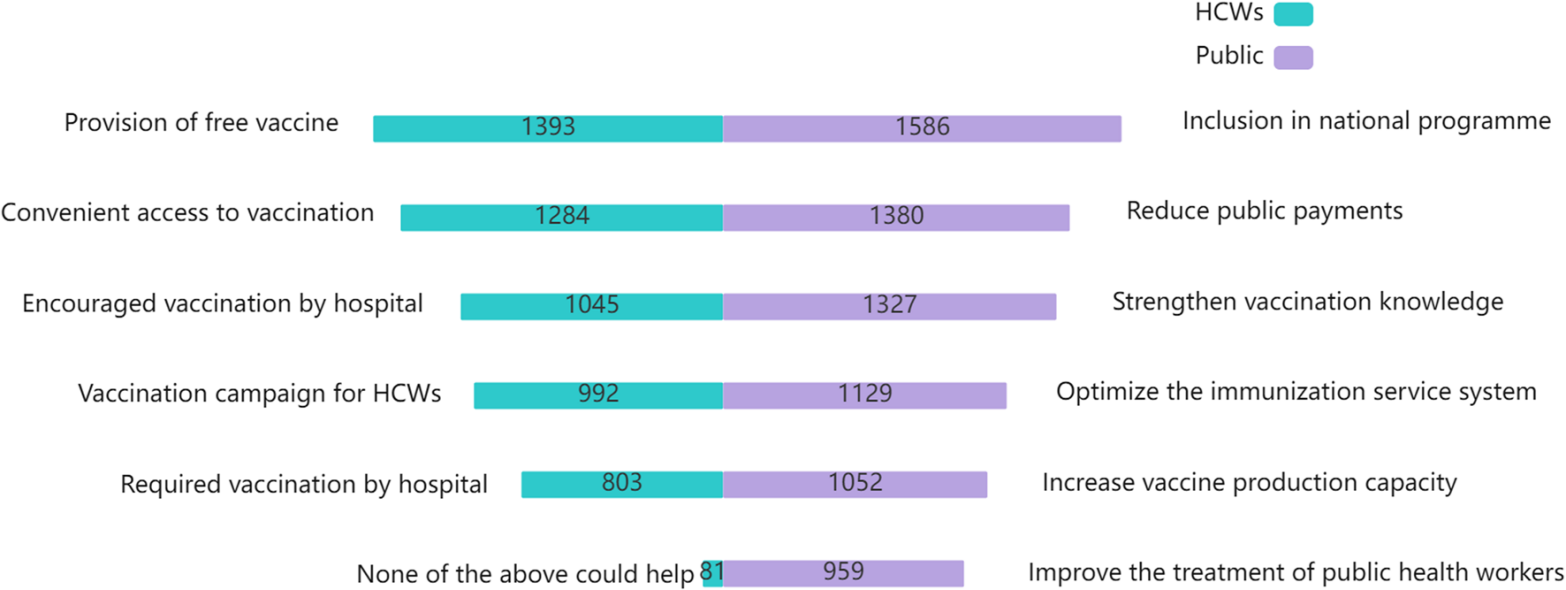
Driving factors for influenza vaccination among healthcare workers and the public^a^. ^a^Numbers represent the number of people who selected that option

To increase the coverage of influenza vaccination among the public, 93.5% of HCWs suggested incorporating influenza vaccination into the national immunization program; 81.3%, reducing self-payment for vaccines; 78.2%, strengthening health education and publicizing knowledge about vaccination; 66.5%, optimizing the immunization service system; 62.0%, increasing vaccine production capacity; 56.5%, improving the treatment of public health practitioners; and 56.3%, increasing investment in public health personnel training (Fig. 2).

## Discussion

HCWs are the priority group for influenza vaccination in China and abroad; however, the domestic influenza vaccination rate had always been low. The respondents of this study were users of the "Breath Circles" platform, most of whom were respiratory physicians or nurses who were aware of the dangers of respiratory diseases. However, the vaccination rate among them in the 2021/2022 influenza seasons was 35.4%, far lower than that in developed countries in Europe and the United States [9]. This may be related to national policies—China has not included influenza vaccination in the immunization program. Only 19.0% of the respondents reported that their workplace offered free vaccination. In addition, except for mandatory vaccination, no single intervention has been shown to rapidly and substantially increase and sustain vaccination uptake [10]. A study found that voluntary policy-based vaccination rarely achieved and maintained a > 40% influenza vaccination rate in practice [11]. In the United States, a large proportion of hospitals mandate HCWs to receive the influenza vaccine (61.4% in 2017) [12]. The Virginia Mason Medical Center in Seattle, USA, uses influenza vaccination as an employment condition, and in 2005, the implementation of a mandatory vaccination policy increased vaccination coverage among more than 5000 HCWs in the next four influenza seasons by > 98% [13].

At the same time, the vaccination rate among HCWs during the 2020/2021 and 2021/2022 influenza seasons in this study was lower than that in the 2019/2020 influenza season (67%) [14], but higher than that in the 2018/2019 influenza season (11.6%) [15]. The reason for the higher rate compared to that of the 2018/2019 influenza season may be due to the official statement by the Chinese Health Commission in 2018/2019 encouraging influenza vaccination. This was the first specific guideline for the vaccination of HCWs put forward, requiring medical institutions at all levels to provide free influenza vaccination to HCWs and ensure that all HCWs in high-risk departments are vaccinated. The reason for the lower rate compared to that in the 2019/2020 influenza season is probably because the COVID-19 pandemic reduced access to vaccines. COVID-19 vaccination became the priority at all level medical facilities, and the influenza vaccine cannot be administered at the same time. The decrease in vaccine coverage among HCWs in the 2021/2022 influenza season compared to the 2020/2021 influenza season may be attributed to the COVID-19 vaccination campaign in key populations in China, which started on December 15, 2020 [16], and was expanded to the general population from February 19, 2021 [17]. The utilization of routine immunization resources by COVID-19 has led to the inconvenience of influenza vaccination, given that coadministration of the two vaccines is not allowed. In addition, the free influenza vaccination campaign in most areas were completed by the end of November in previous years, and the majority of people in other regions had been vaccinated by February. Our study, which completed questionnaire response collection before the end of the influenza season, may have slightly underestimated the vaccine coverage. In addition, our research reported on the economic level of those living in the city, engagement in vaccination work, frequent recommendation of respiratory infectious disease-related vaccines to suitable vaccination populations, supportive attitude for all HCWs to be influenza vaccinated, work place requirement, work place free vaccination policies. HCWs were more likely to be vaccinated if the vaccinations were free. Meanwhile, in this study, the two main reasons why HCWs were vaccinated in 2021/2022 were the HCW’s concerns regarding infecting others and contracting influenza themselves, which was consistent with previous studies elsewhere (Italy [18], Belgium [19], Slovenia [20]), Peru [21], Australia [22], and Singapore [23]).

Vaccine hesitancy among HCWs is a public health challenge [24]. The main reasons why the HCWs in this study were not vaccinated during the 2021/2022 influenza season included inconvenient vaccination locations, which may be caused by the decreasing number of free influenza vaccination facilities due to the impact of the COVID-19 pandemic since more vaccination facilities have been diverted to COVID-19 vaccination, and access the influenza vaccine has decreased.

However, the COVID-19 pandemic has not subsided, and the low influenza vaccination rates among HCWs may cause problems. The high incidence of influenza may cause HCWs to contract influenza and COVID-19, or other respiratory infectious diseases**,** resulting in an epidemic of multiple respiratory infectious diseases. In addition, the influenza vaccine also strengthens immunity and reduces the severity of COVID-19 [25]. The WHO noted in the Global Influenza Strategy 2019–2030 that an outbreak of influenza may highlight the burden and severity of annual epidemics on the global population and health systems of countries; seasonal epidemics may highlight the economic burden of direct and indirect costs [26]. A recent study in the United States showed that mandatory influenza vaccination policies reduced symptom absenteeism rates among HCWs as influenza vaccination rates increased [27]. Influenza vaccination also saves countries costs. A review of more than 140 studies showed that the per capita cost of incidences of seasonal influenza ranged from USD 30 to over USD 60, and that the cost-effectiveness ratios for vaccination ranged from USD 10,000/outcome to more than USD 50,000/outcome [28].

In summary, effective measures should be taken to improve influenza vaccination coverage among HCWs. Our study found that HCWs who were required to be vaccinated by hospitals were more likely to be vaccinated; which is consistent with findings in the United States, where the influenza vaccination rate among HCWs as 92.3% during 2016–2017 [29], and the highest vaccination rates were recorded among HCWs whose employer required influenza vaccination (96.7%), compared to 45.8% in healthcare facilities where influenza vaccination was not required, promoted, or offered on-site. As free vaccination was most likely the driving factor for promoting influenza vaccination among HCWs, hospitals could formulate free vaccination policies to encourage vaccination. In addition, access to influenza vaccination also needs to be improved through measures such as improving the public health function of hospitals and providing influenza vaccination points in hospitals. On-site vaccination is also an effective measure to improve vaccine coverage. An Italian study found that the introduction of an on-site strategy doubled influenza vaccine coverage in the 2017/18 influenza season compared to the previous season [30]. Technical guidelines for influenza vaccination in China (2021–2022) also recommend increasing the number of primary influenza vaccination points, starting vaccination earlier, extending the duration of vaccination, increasing daily service hours, and encouraging influenza vaccination campaigns for HCWs [31]. In addition, since influenza and COVID-19 vaccines cannot be administered at the same time, the current Technical Guidelines for COVID-19 Vaccination (First Edition) in China recommend that the interval between influenza and COVID-19 vaccinations should be > 14 days. However, existing research has not found clear evidence of immunogenicity and safety concerning inactivated influenza vaccines and combining immunization [32]. Future studies could focus on combining immunization regimens, which is important for the prevention and control of the risk of superimposed epidemics in the future.

This study had several limitations. The HCWs in this study had a higher level of education than HCWs in China in general. Therefore, our findings may not represent the vaccination status of HCWs nationwide. However, the low vaccination rates among these highly educated HCWs also reflected the poor vaccination rates among the general population in China. Second, the vaccination status of HCWs in this study was self-reported rather than based on actual vaccination records, which may be affected by recollection bias. In the future, we will continue to track surveyed HCWs, expand the survey population, and focus on the changes in influenza vaccination order to provide a reference for vaccination and influenza prevention and control.

## Conclusions

Influenza vaccination coverage among HCWs in China remained low and showed a downward trend during the COVID-19 pandemic. A free influenza vaccination policy and mandatory workplace vaccination are the factors that drive vaccination. Improving influenza vaccination coverage among HCWs requires further effective measures and the public health function of hospitals should be improved. Hospitals could formulate free vaccination policies and improve the convenience of influenza vaccination through measures such as setting up several influenza vaccination points in hospitals. In addition, combined immunization regimens should be considered in future studies.

## Supplementary Information

**Additional file 1:** Questionnaire

**Additional file 2: Table S1.** Characteristics of HCWs surveyed and in China Health Statistics Yearbook 2021.

## Data Availability

The datasets used and/or analyzed during the current study are available from the corresponding author upon reasonable request.

## Abbreviations

GDP: Gross domestic product
HCWs: Healthcare workers
PLADs: Provincial-level administrative divisions
