# Supplementary material for: Decreased influenza vaccination coverage among Chinese healthcare workers during the COVID-19 pandemic
Source: Infect Dis Poverty. 2022 Oct 8;11:105. doi: 10.1186/s40249-022-01029-0 (PMC9547087; doi:10.1186/s40249-022-01029-0)
Supplement: Supplementary file 1 — Additional file 1: Questionnaire [file 40249_2022_1029_MOESM1_ESM.docx]

# Appendix 1. Questionnaire

## **Ⅰ. Basic information**

1. Your age (in years): (Excluding age <18 or >65 years)

a. < 25 years old

b. 25–34 years old

c. 35–44 years old

d. 45–54 years old

e. 55–59 years old

f. 60 years old and above

2. Your gender?

a. Male

b. Female

3. Your highest education?

a. ≤ Technical secondary

b. Bachelor & Junior college student

c. Postgraduate and above

4. Your province? (Fill in the blanks): ______

5. What is your occupation?

a. Clinician

b. Nurse

c. Medical technician

d. Vaccination staff

e. Others (administration, logistics staff, medical school staff, research institute staff, medical students, etc.)

6. Your working years: By working years

a. < 5 years

b. 5 to 9 years

c. 10 to 19 years

d. 20 to 29 years

e. 30 years and above

7. What is your hospital's category?

a. Primary hospitals

b. Secondary hospitals

c. Tertiary hospitals

8. Which department or division do you work in?

a. Respiratory medicine department

b. Infectious department

c. Pediatrics

d. Emergency medicine department

e. Geriatrics department

f. Gynecology

g. Obstetrics

h. Dermatology

i. Other departments (except internal medicine, surgery and medical technology departments of the above departments)

9. What is your professional title?

a. Above intermediate

b. Intermediate

c. Below Intermediate

d. Unclassified/ Unknown

10. Has your hospital set up a routine vaccination clinic?

a. Yes

b. No

11. Does your daily work involve in vaccination?

a. Yes

b. No

## **Ⅱ. HCWs’ knowledge about vaccination and willingness to get vaccinated**

12. Have you ever taken the initiative to learn about vaccines and health-related knowledge?

a. Yes

b. No

13. How often do you take the initiative to learn about vaccines and health-related knowledge?

a. Once a day

b. Once a week

c. Once a month

d. Once half of a year

e. Once a year 6. None

14. How often do you recommend respiratory infectious diseases’ vaccine to suitable populations?

a. Frequently

b. Occasionally

c. Never

15. Do you support all HCWs to uptake influenza vaccine?

a. Yes

b. No

16. If the vaccination is free, will you uptake influenza vaccine?

a. Yes

b. No

c. Not clear

## **Ⅲ. Workplace vaccination policies**

17. What is your workplace's current policy toward influenza vaccination?

a. Required vaccination

b. Encouraged vaccination (e.g. through health education or health communication)

c. Neither required nor encouraged

d. Not clear

18. Does your workplace currently have free influenza vaccination policies?

a. Yes (free vaccination for all staff)

b. Yes (free vaccination for staff in high-risk department)

c. Do not have free vaccination policy

d. Not clear

## **Ⅳ. Vaccination state and payments**

19. Did you receive the influenza vaccine during the 2020/2021 influenza season?

a. Yes

b. No

20. Have you received the influenza vaccine during 2021/2022 influenza season? (September 2021 to present)?

a. Yes

b. No

21. How did you pay for the influenza vaccination during 2021/2022 influenza season?

a. Self-pay

b. Instant free vaccination

c. Hospital reimbursement after vaccination

d. Medicare reimbursement

e. Other

## **Ⅴ. Reasons for vaccination**

22. What is the main reason for you to receive influenza vaccination this influenza season? (Instructions for filling out: Please select the 3 main reasons you think) [Multiple choice]

a. Worry about contracting influenza

b. Worry about spreading influenza to others

c. Easy access to vaccination from the workplace

d. Required by the workplace

e. Recommendations from the national policy-making body

f. Previous experience with vaccination

g. Free vaccination

f. Preventing/reducing absenteeism from work

23. What is the main reason you did not get the influenza vaccine this influenza season? (Instructions for filling out: Please select the 3 main reasons you think) [Multiple choice]

a. Do not know where to get vaccinated

b. Do not know when to get vaccinated

c. Too busy in work

d. Influenza will not cause severe illness

e. Worry about the adverse reactions of influenza vaccine

f. Influenza vaccine is not effective in prevention

g. Inconvenient location for vaccination

h. Vaccines are not free

i. Having contraindications

j. Pregnant or lactating women

24. Which of the following initiatives do you think will motivate HCWs to uptake influenza vaccine? [Multiple choice]

a. Free vaccination

b. Convenient access to vaccination

c. Encouraged vaccination by hospital

d. Required vaccination by hospital

e. Vaccination campaign for HCWs

f. None of the above measures could help

25. What suggestions do you have for improving influenza vaccination coverage to public? [Multiple choice]

a. Inclusion influenza vaccination in national programme

b. Reduce public payments

c. Strengthen the vaccination knowledge

d. Optimize the immunization service system

e. Increase vaccine production capacity

f. Improve the treatment of public health workers
