# Supplementary material for: Decreased influenza vaccination coverage among Chinese healthcare workers during the COVID-19 pandemic
Source: Infect Dis Poverty. 2022 Oct 8;11:105. doi: 10.1186/s40249-022-01029-0 (PMC9547087; doi:10.1186/s40249-022-01029-0)
Supplement: Supplementary file 2 — Additional file 2: Table S1. Characteristics of HCWs surveyed and in China Health Statistics Yearbook 2021. [file 40249_2022_1029_MOESM2_ESM.docx]

**Table S1.** Characteristics of HCWs surveyed and in China Health Statistics Yearbook 2021.

| **Characteristic** | **Category** | **HCWs in China (%)** | **HCWs in this study (%)** | ***P* - value for chi-square test** |
| --- | --- | --- | --- | --- |
| Gender |  |  |  | 0.31 |
|  | Male | 27.6 | 34.5 |  |
|  | Female | 72.4 | 65.5 |  |
| Age, years |  |  |  | 0.15 |
|  | < 25 | 8.9 | 6.2 |  |
|  | 25–34 | 40.0 | 32.6 |  |
|  | 35–44 | 24.8 | 40.2 |  |
|  | 45–54 | 16.5 | 16.9 |  |
|  | 55–59 | 4.7 | 3.1 |  |
|  | ≥ 60 | 5.1 | 1.0 |  |
| Years of working |  |  |  | 0.21 |
|  | < 5 | 24.9 | 17.7 |  |
|  | 5–9 | 23.1 | 19.2 |  |
|  | 10–19 | 23.1 | 36.4 |  |
|  | 20–29 | 16.2 | 18.8 |  |
|  | ≥ 30 | 12.7 | 8.0 |  |
| Education degree |  |  |  | < 0.0001 |
|  | Postgraduate | 5.9 | 23.6 |  |
|  | Bachelor & Junior college | 74.6 | 74.7 |  |
|  | ≤ Technical secondary school | 19.4 | 1.7 |  |
